# The effect of physiological changes in estradiol hormone during the follicular phase of the menstrual cycle in healthy women on retinal ganglion cell function, retinal and optic nerve vascularization and structure

**DOI:** 10.1007/s10633-026-10105-x

**Published:** 2026-04-25

**Authors:** Mualla Hamurcu, Burcu Polat Gültekin, Melisa Göçmen, Zarife Nurbanu Mendi, Zehra Kurdoğlu

**Affiliations:** 1 Department of Ophthalmology, Ankara Bilkent City Hospital, Ankara, Turkey; 2 Department of Ophthalmology, Izmir City Hospital, Izmir, Turkey; 3 Department of Obstetrics and Gynecology, Ankara Bilkent City Hospital, Ankara, Turkey

**Keywords:** Electrophysiology, Menstrual cycle, Estradiol, Optical coherence tomography, Optical coherence tomography angiography, Pattern electroretinogram, Retinal nerve fiber layer thickness

## Abstract

**Purpose:**

This study aimed to investigate the effects of estradiol changes occurring during the early follicular phase of the menstrual cycle on retinal ganglion cell function, retinal and optic nerve vascularity and structure in healthy adults using pattern electroretinography (PERG), optical coherence tomography (OCT), and optical coherence tomography angiography (OCTA).

**Methods:**

The study included 26 healthy subjects with a visual acuity of 1.0. Serum hormone levels, intraocular pressure (IOP), cycloplegic spherical equivalent (SE), PERG, retinal nerve fiber layer (RNFL) thickness and OCTA measurements were evaluated. PERG tests were performed using the Metrovision Monpack system. Spectral-domain OCT (Topcon Corporation, Tokyo, Japan); OCTA (Optovue, Inc, Fremont, CA) devices were used. All tests were conducted on the 1st and 14th days of the menstrual cycle, and the results were compared.

**Results:**

The mean serum estradiol values on days 1 and 14 were 51 ± 19 ng/L and 304 ± 50 ng/L, respectively. PERG revealed no statistically significant differences in the amplitude or latency of the P50 and N95 waves between days 1 and 14. RNFL thickness did not differ significantly in the mean, superior, or inferior quadrants (*p* = 0.90, *p* = 0.85, and *p* = 0.39, respectively). OCTA analysis showed that peripapillary and macular vascular density values were similar between days 1 and 14 (*p* > 0.05). Correlation analysis revealed no significant relationship between electrophysiological parameters, RNFL thickness measurements, and retinal vascular densities (*p* > 0.05).

**Conclusion:**

In healthy women, no significant functional, vascular, or structural differences were observed in PERG, OCT, or OCTA parameters between low and high estradiol levels on days 1 and 14 of the menstrual cycle. Unlike the persistently low estradiol levels seen in menopause, physiological estradiol fluctuations during a healthy menstrual cycle do not cause functional, structural, or vascular changes in the optic nerve or retina.

## Introduction

The menstrual cycle is a complex physiological process characterized by rhythmic fluctuations of sex steroids, which exert their effects through specific receptors distributed throughout various ocular tissues Ovarian steroids influence the central nervous system, including sensory processing centers, by shaping the dorsal and ventral visual streams during development [1, 2]. Studies have demonstrated the presence of sex steroid hormone receptors in various ocular tissues, including the cornea, iris, ciliary body, lens, conjunctiva, retina, and the lacrimal and meibomian glands [2, 3]. It has been suggested that sex steroids modulate the morphology of the optic nerve head, with some evidence indicating that the neuroretinal rim area may decrease during phases with distinct hormonal profiles, while the cup area increases [4]. While variations in corneal thickness and intraocular pressure (IOP) have been documented throughout the cycle, the potential impact of the estradiol peak on macular and optic nerve function remains less clear [5–7]. As in our study, the specific effect of estradiol on retinal vascularity and neuroprotection can be investigated with high sensitivity through a multifaceted evaluation including OCT, OCTA, and PERG methods [8].

The first 14 days of the menstrual cycle constitute the follicular phase, where follicle-stimulating hormone (FSH) levels begin to rise from the beginning of menstruation. Serum estradiol levels gradually rise as developing follicles produce increasing amounts of estradiol. Once estradiol reaches its peak, FSH is suppressed through a negative feedback mechanism. The estradiol peak then triggers a surge in luteinizing hormone (LH), leading to ovulation approximately 24 h later [9]. Considering these hormonal dynamics, we selected the period of lowest and highest estradiol in both the early and late follicular phases to evaluate the effects of estradiol on ocular parameters. This design allowed us to more clearly evaluate the impact of physiological estradiol fluctuations on ocular structures by minimizing the potential impact of progesterone.

There are also studies on the long-term decline in estradiol levels during menopause and the effects of postmenopausal hormone therapy. Postmenopausal hormone therapy has been shown to increase retinal blood flow and protect the retinal nerve fiber layer [10]. OCTA-based studies have likewise demonstrated variability in retinal hemodynamics across the menstrual cycle in healthy women [8, 10].

Spectral-domain OCT provides high-resolution cross-sectional imaging of retinal structures. OCTA, by providing high-resolution, depth-resolved imaging of the retinal microvasculature, enables a detailed and quantitative assessment of vascular density across capillary plexuses. The electrophysiological test PERG evaluates macular and ganglion cell function by measuring the visual system’s response to patterned stimuli. Through this multimodal approach, the potential impact of estradiol on blood-retina barrier regulation, retinal vascularity, neuroprotection, and the pathophysiology of various neuro-ophthalmic disorders can be investigated.

In this study, we aimed to evaluate the effects of physiological estradiol fluctuations on retinal and optic nerve function, vascularity and structure during the menstrual cycle using OCT, OCTA and PERG.

## Participants and methods

This prospective study included 26 eyes of 26 healthy female volunteers aged 20–33 years who had regular menstrual cycles, no history of childbirth and no history of hormone therapy [10]. PERG tests were performed binocularly in accordance with ISCEV standards to ensure fixation stability and minimize patient fatigue [11]. To avoid inter-eye correlation bias, only the right eye was used for statistical analysis [12]. Each participant provided written informed consent, and the study was approved by the Institutional Ethics Committee of Ankara Bilkent City Hospital. All procedures adhered to the principles of the Declaration of Helsinki.

All participants underwent a comprehensive ophthalmologic examination, including best-corrected visual acuity (BCVA), anterior and posterior segment biomicroscopy, and IOP measurement. Individuals with a BCVA of 1.0 or better in both eyes and a spherical equivalent (SE) refractive error between – 2.50 and + 2.50 diopters were included. PERG tests were performed binocularly in the hospital’s certified electrophysiology laboratory. Following PERG recordings, OCT and OCTA examinations were conducted, and all data were collected. Each participant was tested twice, on the 1st and 14th days of the menstrual cycle.

Participants had no systemic comorbidities, had not experienced abortion or childbirth within the last three months, were not using oral contraceptives, hormone therapy, or vasoactive medications, and had no history of ocular surgery. Individuals with ophthalmologic or systemic diseases, irregular menstrual cycles, premature ovarian insufficiency, polycystic ovary syndrome, or a menstrual cycle length outside the 21–35 day range during the last three cycles were excluded. Mean serum estradiol values were measured on days 1 and 14.

## Electrophysiological tests

PERG was conducted using a commercial electrophysiology device (Monpack, Metrovision, France) with Hawlina-Konec loop-type recording electrodes, adhering to the standards of the International Society for Clinical Electrophysiology of Vision (ISCEV) [11]. Active electrodes were carefully placed on the bulbar conjunctiva without pupil dilation, while reference electrodes were positioned near the bilateral lateral canthus.

For each participant, a square size of 0.8 degrees was used for PERG. The stimulus screen had a constant mean luminance of 50 cd/m^2^, with a pattern contrast set at 95%. The pattern rotated around the fovea at a rate of 4.5 reversals per second within a 15º stimulus area. All stimulus parameters, including luminance and contrast, were calibrated by the company. Binocular PERG was recorded after correcting for refractive error to achieve best-corrected visual acuity. The P50 amplitude was measured from the base of N35 to the peak of P50, while the N95 amplitude was measured from the peak of P50 to the base of N95. Latency and amplitude calculations for the P50 and N95 waves were documented.

## OCT and OCTA ımaging

All participants underwent imaging with spectral-domain OCT (Topcon Corporation, Tokyo, Japan). Following pharmacological pupil dilation, imaging was performed twice by the same examiner during the same visit for each scanning protocol. The macular cube 512 × 128 protocol and the optic disc 200 × 200 protocol were applied. For the RNFL protocol, a 360-degree circular scan with a 3.4-mm diameter centered on the optic disc and an 8 × 8 mm volumetric optic disc scan were obtained. Only well-centered images with a signal strength of 7/10 or higher were selected for analysis. The RNFL thickness analysis software calculated total and quadrant-spesific mean RNFL thicknesses.

OCTA (Optovue Inc., Fremont, CA) imaging was performed using the split-spectrum amplitude decorrelation angiography (SSADA) algorithm. Volumetric scans were acquired at 304 × 304 A scan size with a scanning speed of 70,000 A-scans per second. Retinal vascularity was assessed using 4.5 × 4.5 mm scans in the peripapillary region and 3 × 3 mm scans in the macular region. Peripapillary vessel density and superficial (SCP) and deep (DCP) capillary plexuses were evaluated using automated segmentation generated by the software.

Peripapillary vessel density was calculated at the retinal pigment cell (RPC) layer, defined as the area between the outer border of the RNFL and the internal limiting membrane (ILM). The SCP was defined with its inner border 3 µm below the ILM and its outer border 15 µm below the inner plexiform layer (IPL). The DCP layer was defined with its inner border 15 µm below the IPL and its outer border extending up to 70 µm below the IPL. Vessel densities were calculated as the ratio of vessel pixels to the total number of pixels. Images with a signal strength index below 40 or those affected by motion artifacts were excluded from analysis. The foveal center was verified by aligning the OCT scans with the OCTA images.

## Data analysis

Statistical analysis was performed using SPSS version 21.0 (SPSS Inc., Chicago, IL, USA). The Shapiro–Wilk test was used to assess the normality of the data distribution. Comparisons between values on the 1st and 14th days of the menstrual cycle were conducted using the paired-samples Wilcoxon test, while correlations were analyzed using the Spearman rank correlation test. Statistical significance was set at *p* < 0.05.

## Results

A total of 26 female participants were included in the study. The mean age of the participants was 25 years (range: 20–33 years). The anterior and posterior segment examinations and intraocular pressure measurements of the participants were within normal limits. The mean serum estradiol levels on days 1 and 14 were 51 ± 19 ng/L and 304 ± 50 ng/L, respectively. Table 1 presents the median values and minimum–maximum ranges of the PERG components on days 1 and 14 of the menstrual cycle. No statistically significant differences were found in the amplitudes and latencies of the P50 wave during the follicular phase (*p* = 0.74 and *p* = 0.88, respectively). Similarly, no statistically significant differences were observed in the amplitudes and latencies of the N95 wave (*p* = 0.98 and *p* = 0.8, respectively).

**Table 1:** Comparison of PERG values according to the 1st and 14th day of the menstrual cycle

Parameter	First day of menstruation	14th day of menstruation	*p* value
P50 wave			
Peak time (ms)	48.4 ( 43.3–56.6)	48.4 (44.2–54.9)	0.88
Amplitude (µV)	9.1 (3.7–16.3)	9.0 (3.0–13.5)	0.74
N95 wave			
Peak time (ms)	99.9 (83.2–121)	98.9 (64.6–124)	0.83
Amplitude (µV)	10.9 (5.6–19.1)	10.8 (3.0–16.8)	0.98

^*^Values are Median (Min–Max), Paired samples Wilcoxon test

According to Table 2, no significant differences were detected in average, superior, or inferior RNFL thickness between days 1 and 14 of the menstrual cycle (*p* = 0.90, *p* = 0.85 and *p* = 0.39, respectively).

**Table 2:** Comparison of retinal nerve fiber layer thickness measurements according to the 1st and 14th days of the menstrual cycle

RNFL thickness (quadrant, µm)	First day of menstruation	14th day of menstruation	*P* value
Average	118.1 (104–148)	115.6 (105–135)	0.90
Superior	118.2 (99–118.2)	118.7 (99–141)	0.85
Inferior	119.8 (106–153)	115.6 (100–129)	0.39

^*^Values are Median (Min–Max), Paired samples Wilcoxon test

Table 3 displays peripapillary vessel density values and macular SCP and DCP vascular densities. OCTA analysis showed no statistically significant differences in vascular densities between days 1 and 14 (*p* > 0.05).

**Table 3:** Comparison of OCTA values according to the 1st and 14th days of the menstrual cycle

Vessel density (%)	First day of menstruation	14th day of menstruation	*p* value
Peripapillary			
Total	49.6 (43.9–52.8)	48.9 (40.1–54)	0.71
Disc	48.1 (40–58.5)	47.4 (39.9–58.5)	0.41
Peripapillary	53 (47–57.4)	51.1 (38.3–57.4)	0.15
Superior hemispher	52.7 (46–58.4)	50.7 (25.3–58.4)	0.39
Inferior hemispher	53.2 (47.1–57.1)	52 (41.5–56.2)	0.17
Macula			
Superficial capillary layer			
Total	47.8 (37.6–55.6)	47.6 (37.7–53.5)	0.51
Foveal	17.4 (6.8–27.3)	17.5 (9.8–30.2)	0.91
Parafoveal	49.1 (35.4–57.8)	48.7 (35.4–56.6)	0.59
Perifoveal	48.4 (39.1–57.1)	48.3 (39.1–57.1)	0.46
Deep capillary layer			
Total	47.8 (36.3–57.2)	47.4 (36.2–57.8)	0.86
Foveal	35.9 (25.1–58.3)	36.3 (24.5–58.3)	0.86
Parafoveal	54.0 (43.3–60.6)	53.7 (44.4–59.3)	0.91
Perifoveal	47.5 (15–59.6)	47.1 (15–58.2)	0.8

^*^Values are Median (Min–Max), Paired samples Wilcoxon test

Correlation analysis revealed no significant associations between electrophysiological parameters, RNFL measurements and retinal vascular densities (*p* > 0.05).

## Discussion

In our study, which aimed to evaluate the physiological fluctuations of estradiol between days 1 and 14 of the follicular phase, independent of the effect of progesterone, no significant difference was observed in PERG, OCT, or OCTA parameters between days 1 and 14 of the menstrual cycle; this indicates that physiological estradiol fluctuations do not lead to measurable functional, vascular, or structural changes in healthy women.

Sex hormones play an important role not only in the reproductive system but also in many other organs and tissues that appear anatomically similar in men and women [9]. Depending on the target site, these hormones may act through endocrine, paracrine or autocrine mechanisms. Estradiol, in particular, have been shown to exert numerous beneficial ocular effects, including a reduction in IOP and a supportive influence on the lamina cribrosa [3, 9, 12, 13].

Nuzzi et al. described the protective and modulatory actions of estradiol on various ocular structures, highlighting its potential neuroprotective, vascular, and biomechanical effects on the optic nerve head and retinal tissues. Estrogens, have been shown to exert neuroprotective effects on the retina by influencing neuronal survival, synaptic activity, and oxidative stress pathways. They also play a role in modulating ocular blood flow through vascular endothelial interactions and regulation of nitric oxide production. Notably, structural and functional differences have been reported between the male and female retina, and fluctuations or deficiencies in sex hormones may influence susceptibility to or progression of specific ocular diseases [9, 12, 13].

PERG is a practical and non-invasive ocular electrophysiological test that allows us to objectively evaluate macular and ganglion cell function [11]. In our study, no significant difference was observed in PERG components, especially P50 and N95 amplitudes and latency times, on the 1st and 14th day of the follicular phase. This suggests that fluctuations in estradiol levels do not affect optic nerve and retina functions in the physiological process. Although there are studies on the chronic decline of postmenopausal estradiol or postmenopausal hormone replacement, our study is distinctive in that it evaluates the effects of estradiol fluctuations in the physiological process [8, 10].

OCT-based studies have reported a significant decrease in subfoveal choroidal thickness during the midluteal phase, as demonstrated using spectral-domain OCT [6]. Our study, however, focused on acute physiological fluctuations in the follicular phase of the menstrual cycle in young, healthy women. While Ataş et al. demonstrated the effects of long-term hormonal depletion, our findings showing no significant change in macular vascular density or retinal structure between days 1 and 14 suggest a strong homeostatic resistance of the neurovascular unit to transient estradiol surge. This explains the hormonal physiological cycle; while estrogen deprivation in menopause can lead to atrophy, the physiological surge during the reproductive cycle does not lead to changes in retinal vascularity, structure, and ganglion cell function [14]. Similarly, Akar et al. examined 38 healthy women with a mean age of 25.6 ± 3.7 years (range, 21–34 years). Blood estradiol levels were significantly lower in the late luteal phase (35.8 pg/mL). The neuroretinal rim area decreased significantly during the luteal, while the linear cup-to-disc ratio, cup-to-disc area ratio, and cup area were significantly higher in the luteal phase. These findings suggest that sex hormone fluctuations during the normal menstrual cycle may significantly affect neuroretinal rim area and cup morphology of the optic nerve head [4]. In contrast, Alpogan et al. reported no significant differences in RNFL or GCC (ganglion cell complex) parameters between reproductive-age and postmenopausal women after adjusting for age and BMI [15]. Consistent with these findings, in our study, we also observed no significant differences in average, superior, or inferior RNFL thickness during the follicular phase.

OCTA studies have described menstrual cycle related and hormonally driven changes in ocular microvasculature. Donica et al. reported that, in 22 eyes of healthy reproductive-age women with a regular menstrual cycles, choroidal thickness in the parafoveal and subfoveal regions was significantly increased during the ovulatory phase compared with the early follicular phase [9]. In postmenopausal women, an increase in both superficial and deep foveal avascular zone (FAZ) areas, and a decrease in choroidal blood flow have also been documented [16]. Toker et al. demonstrated that estradiol and testosterone significantly influence retrobulbar blood flow velocities and resistive indices, further highlighting the vascular effects of estradiol [17]. Additionally, Guo et al. observed alterations in the deep capillary plexus during the ovulatory phase [18]. Ozbek et al. reported that significant differences in choriocapillaris vessel density were observed across early follicular, ovulatory, and mid-luteal phases within the peripapillary region, particularly in the temporal and inferior quadrants [19]. However, in our study, no significant differences were observed in OCTA parameters during the follicular phase.

In our study, RNFL thickness measurements did not show any significant difference in the follicular phase. OCTA parameters, including peripapillary vessel density and macular SCP and DCP vessel densities, were also stable throughout the phase. These findings indicate that physiological changes in estradiol levels in the follicular phase of a normal menstrual cycle do not cause significant differences in retina and optic nerve structure and vascularization and do not significantly alter microvascular perfusion in healthy women.

In the study by Brûlé et al., it was shown that higher estrogen levels can increase the a and b wave amplitudes in full-field ERG [20]. The reason for not detecting any difference in our study may be due to the fact that PERG specifically evaluates ganglion cell function. Therefore, full-field ERG may also have a different sensitivity compared to the retinal response obtained [21, 22]. The neuroprotective potential of estradiol has been demonstrated through experimental models. Yamashita et al. showed that estradiol improved electroretinographic responses in diabetic female rats and had functional and structural protective effects against N-methyl-D-aspartate-induced retinal neurotoxicity. These studies highlight the role of estrogen in improving or protecting retinal function in damaged tissues [23, 24]. In our study, our findings show that physiological estrogen fluctuations in healthy adult women do not cause functional and structural changes, but maintain their condition. The ‘protective’ effect of the increase in estradiol during the follicular phase in the healthy retina supports the stability observed in our PERG and OCTA parameter’s. In addition, focusing on the follicular phase allowed us to isolate the effects of estradiol from progesterone [25]. Recent research highlights variability in ocular hemodynamics but our findings show that these fluctuations do not significantly affect clinical measurements of the optic nerve and retina in a healthy follicular cycle [8, 19].

Studies with larger sample sizes can be planned to better understand the relationship between ocular parameters and serum hormone concentrations in specific ocular and systemic conditions. This study may form the basis for future research with larger groups to clarify the effects of hormonal fluctuations on ocular parameters. Additionally, evaluation of the role of hormones in optic nerve and retina diseases may facilitate diagnosis and treatment planning and management.

## Data Availability

No datasets were generated or analysed during the current study.

## Declarations

### Statement on the Welfare of Animals

This article does not contain any studies with animals performed by any of the authors.
